# Medical school origins of award-winning physicians; analysis of a complete national dataset

**DOI:** 10.1186/s12909-024-05200-z

**Published:** 2024-03-08

**Authors:** Sinclair Steele, Gabriel Andrade, Nisha Shantakumari, Debadatta Panigrahi

**Affiliations:** College of Medicine, Ajman University, University Street, Al Jerf 1, Ajman, UAE

**Keywords:** Physicians, Medical schools, Medical careers, Award-winners, Globalization, International medical graduates

## Abstract

**Background:**

Educators and medical students share the same objective of achieving success in medical practice. Both groups consider doctors’ successes to include optimum patient care outcomes and positive career progressions. Accordingly, identifying common educational features of such high-achieving doctors facilitates the generation of excellence amongst future medical trainees. In this study we use data from the British clinical merit award schemes as outcome measures in order to identify medical school origins of doctors who have achieved national or international prominence.

**Methods:**

Britain has Clinical Excellence Awards/Distinction Awards schemes that financially reward all National Health Service doctors in England, Scotland and Wales who are classified as high achievers. We used these outcome measures in a quantitative observational analysis of the 2019-20 dataset of all 901 national award-winning doctors. Where appropriate, Pearson’s Chi-Square test was applied.

**Results:**

The top five medical schools (London university medical schools, Glasgow, Edinburgh, Oxford and Cambridge) were responsible for 51.2% of the physician merit award-winners in the 2019-20 round, despite the dataset representing 85 medical schools. 91.4% of the physician merit award-winners were from European medical schools. The lowest national award-winners (tier 3) originated from 61 medical schools representing six continents. International medical graduates comprised 11.4% of all award-winners.

**Conclusions:**

The majority of physicians who were national merit award-winners originated from only five, apparently overrepresented, UK university medical schools. In contrast, there was a greater diversity in medical school origin among the lower grade national merit awards; the largest number of international medical graduates were in these tier 3 awards (13.3%). As well as ranking educationally successful university medical schools, this study assists UK and international students, by providing a roadmap for rational decision making when selecting physician and non-physician medical education pathways that are more likely to fulfil their career ambitions.

## Background

Outside the Americas, the word “physician” is usually used to refer to an individual who practises internal medicine or related disciplines. Accordingly, it is commonly used in opposition to “surgeon” and it is this use of the word *physician* that applies in the UK and to our study. Understandably, students or trainee doctors that are interested in becoming well-trained physicians seek medical schools that have robust embedded training in the full range of a physician’s skills. Consequently, one measure of the effectiveness of medical education is the ultimate production of such successful doctors. Accordingly, our project examines the educational backgrounds of successful practising physicians in Britain.

Historically, in Britain there have been two clinical merit award schemes established to reward successful clinicians employed in the National Health Service (NHS):

(i) The Clinical Excellence Awards (CEA) scheme, covering Wales and England [1].
(ii) The Distinction Awards (DA) scheme, covering Scotland [1].

The schemes are similar in aims and organization; both offer tiers of local and national awards to high-achieving doctors. However, the CEA scheme is currently being restructured, renamed and re-established as the National Clinical Impact Awards (NCIA), whilst the DA scheme remains in place in Scotland. The doctors receiving such awards gain benefits not only from the effects of these honours on their reputations and career progressions but also from the recurring financial rewards accompanying such accolades [1].

These UK national award schemes were envisioned and implemented after World War II for the pragmatic purpose of motivating senior clinicians to support the newly-created NHS. Since their inception these schemes and their implementation have been the cause of vigorous debate in the UK medical community. As a result, these clinical merit awards have been evaluated and discussed from the perspective of award objectivity [2], specialty distribution [3], regional distribution [3], gender parity [1], age distribution [4] and ethnicity/racial distribution [5] but, until our research series, *not by medical school of origin*. These constructive criticisms have resulted in iterative revisions of these award schemes over the previous three decades. Many medical commentators agree that there should be a system to reward high-achieving clinicians [6] and the CEA/DA/NCIA merit awards are seen as national recognition of clinical career success - accounting for their continuing value, greater than 70 years after their inception. This original innovative research study is part of a series that contributes to the medical education discussion by relating the physician and non-physician merit award-winners to their *medical schools of origin*. We place our findings in the contexts of educational, career and global implications for ambitious prospective medical students, undergraduate medical students and doctors aspiring to attain career success [7, 8].

## Methods

The lists of physician award-winners and non-physician award-winners were retrieved from the source material of the DA annual report (Scotland) for 2019–2020 [9] together with the CEA annual report (England and Wales) [10] for the 2019–2020 awards round. These lists were summations of both the newly selected awardees and the previous award-winners who retained their awards. The medical schools of origin were identified by using the published Medical Register, UK [11] as well as the published Dental Register, UK [12–14].The total number of award-winners was 901 - the university medical schools of origin were successfully identified for 99.8% of these clinicians [13, 14]. Accordingly, 899 doctors were included in the analyzed dataset. Award-winning doctors in the publications above, who were designated as specializing in any of the medical disciplines, were included in this study [13, 14]. In the 2019-20 award round the following specialties were included: general medicine, medicine, acute internal medicine, geriatric medicine, genitourinary medicine, general practice, occupational medicine, respiratory medicine, cardiology, clinical oncology, endocrinology and diabetes, academic general practice, forensic psychiatry, neurology, paediatrics and psychiatry of learning disability [13, 14].

The rankings of medical schools by number of merit award-winning alumni were determined by summation of the number of physician award-winners of A plus (A^+^), A or B grade (or platinum, gold, silver or bronze award-winners) [13, 14]. Only these national level Clinical Excellence Awards and Distinction Awards were included in this study [13, 14]. Combining these parallel and similar award gradings, permitted all of Britain’s (England, Wales and Scotland) excellence award-winners to be analyzed together [13, 14]. As part of our analysis of the grades of awards we collated the award categories to explicitly show the three tiers of national merit awards; A plus and platinum award-winners were combined to yield the top tier (tier 1) of national physician awards [13, 14]. The A and gold awards were combined to create the intermediate tier (tier 2) of national physician awards [13, 14]. Finally, the B and silver/bronze awards were combined to create the lowest tier (tier 3) of national physician merit awards [13, 14]. The same approach was taken with the non-physician data [13, 14].

The rankings of the medical schools by the number of merit award-winning alumni were approximately size corrected by dividing the total number of award-winners that were alumni of the medical school by the number of admissions to the undergraduate medical school in the 2019-20 academic year [13, 14]. We used this pragmatic approach to estimate the size correction rather than the more ideal but inaccessible integral of medical school graduation numbers against time for approximately the last 50 years [13, 14]. The comparison of the distributions of award-winners (physician merit award-winners versus non-physician merit award-winners) was quantified using Pearson’s Chi-Square test with the significance level set to *p* < 0.05 [13, 14]. All procedures were performed in compliance with the pertinent guidelines [13, 14]. Patients and public involvement; no patient involvement [13, 14]. The methods that were applied in our study, and that cover the description in this methods section, were similar to and closely derived from earlier publications in this series, which we cite here [13, 14].

## Results

There were 420 physician merit award-winners in the 2019-20 award round and the largest category was designated “medicine” amounting to 44.1% of all the merit award-winning physicians.

Table 1 shows the ten medical schools that attained the largest number of alumni merit award-winners; these award-winners possessed platinum, gold, silver, bronze, A plus, A or B awards. In addition, Table 1 compares the originating medical schools of the physician and non-physician merit award-winners for the ten medical schools with the largest numbers of award-winners; the table contrasts the numbers and percentages of physician award-winners and non-physician award-winners which the graduates of each medical school attained. Pearson’s Chi-Square test demonstrated a statistically significant difference between the distributions of the medical schools of origin for physician merit award-winners versus the non-physician merit award-winners, *p* < 0.01 (Chi square 32.27, P-value 0.0002). Graduates of London university medical schools, Glasgow, Edinburgh, Oxford and Cambridge medical schools accounted for 51.2% of physician award-winners. In comparison, 53.0% of the non-physician merit award-winners were graduates of five British medical schools: Aberdeen, Edinburgh, Glasgow, London university medical schools and Cambridge.

**Table 1:** Top 10 medical schools; analysis by number of physician award holders, number of non-physician award-winners and total number of award-winners (2019-20)

Medical school	Total number of award -winners	Number of physician award-winners	Percentage of all physician award- winners	Number of non-physician award-winners	Percentage of all non-physician award-winners
London	179	107	25.5	72	15.0
Glasgow	113	50	11.9	63	13.2
Edinburgh	84	35	8.33	49	10.23
Aberdeen	60	13	3.10	47	9.81
Oxford	45	23	5.48	22	4.59
Cambridge	43	20	4.76	23	4.80
Manchester	38	16	3.81	22	4.59
Birmingham	29	18	4.29	11	2.30
Dundee	29	16	3.81	13	2.71
Nottingham	26	13	3.10	13	2.71

Table 2 displays the effect of the approximate medical school size correction on the ranking of the medical schools by number of alumni award-winners. London’s number one ranking (physicians) before size correction dropped to a number six ranking after size correction. Similarly, London’s number one ranking (non-physicians) before size correction became a number nine ranking after size correction.

**Table 2:** Top 10 medical school rankings by number of graduate merit award-winners; with or without size correction (2019-20)

Medical school	Total number of physician award- winners	Ranking by number of physician award- winners	Ranking by physician award- winners after size correction	Total number of non-physician award- winners	Ranking by number of non-physician award- winners	Ranking by non-physician award- winners after size correction
London	107	1	6	72	1	9
Glasgow	50	2	1	63	2	1
Edinburgh	35	3	2	49	3	2
Aberdeen	13	9=	8	47	4	3
Oxford	23	4	3	22	6=	4
Cambridge	20	5	5	23	5	5
Manchester	16	7=	10	22	6=	7
Birmingham	18	6	7	11	10	10
Dundee	16	7=	4	13	8=	6
Nottingham	13	9=	9	13	8=	8

Our analysis included a comparison of the physician A plus/platinum award-winners (designated tier 1) with A/gold award-winners (designated tier 2) and B/silver/bronze award-winners (designated tier 3). The tier 1 physicians award-winners came from 12 medical schools: Birmingham, Cambridge, Dundee, Edinburgh, Gdansk, Glasgow, Ireland (Royal College of Surgeons), London university medical schools, Manchester, Nottingham, Oxford and Sheffield. The tier 2 physician award-winners came from 17 medical schools: Aberdeen, Bangalore, Birmingham, Cambridge, Cologne, Dundee, Edinburgh, Glasgow, Harvard, London university medical schools, Madras, Manchester, Newcastle, Nottingham, Oxford, Sheffield and Southampton. The tier 3 physician award-winners originated from 61 medical schools.

Table 3 contrasts the continental locations of the originating medical schools for physician and non-physician merit award-winners; for the ten medical schools with the greatest numbers of award-winners. 91.4% of physician merit award-winners were from European medical schools, in comparison 91.9% of the non-physician award-winners were from European medical schools. Pearson’s Chi-Square test indicated that there was not a statistically significant difference between the continental locations of the originating medical schools for physicians and non-physician merit award-winners, *p* > 0.05.

**Table 3:** A geographical comparison of the medical schools of origin of physician and non-physician merit award-winners (2019-20)

Continental location of medical school	Non-Physicians			Physicians	
	Total number of non-physician award- winners	Percentage of total number of non-physician award-winners		Total number of physician award-winners	Percentage of total number of physician award-winners
Africa	11	2.30		8	1.90
Asia	22	4.59		19	4.52
Australasia	3	0.63		6	1.43
Europe	440	91.9		384	91.4
North America	3	0.63		2	0.48
South America	0	0.00		1	0.24
Total	479	100%		420	100%

11.9% of all the physician award-winners were international medical graduates (IMGs) - meaning that they were not graduates of UK or Irish medical schools. 10.7% of the non-physician award-winners were IMGs. The physician tier 3 award-winners included the greatest proportion of IMG award-winners at 13.3%.

## Discussion

### Physician merit awards and UK medical schools

Our study is part of the first series to comprehensively analyze British clinical merit award-winners’ medical schools of origin. This project identifies medical schools that have facilitated the successful medical education of physicians by using the outcome measure of clinical merit award-winning. As a result, the data and analysis we provide will be of significance to local potential medical students as well as current and future graduates of International Medical Programs [15]. Our series of studies are the first to *rank medical schools by the number of merit award-winners* originating from each school, and accordingly will provide a new perspective for medical educators.

The UK has long been known to attract international medical graduates to practise medicine. This was further confirmed and quantified in the General Medical Council 2019 workforce study that stated “For the first time, more non-UK medical graduates took up a licence to practise than UK medical graduates.“ [16] As a result of such workforce migrations, the scope of possible medical schools of origin of merit award-winners has essentially become global. Specifically, our database of merit award-winners covering the 2019-20 round has 85 different medical schools represented. This study shows that after being chosen by a “transparent and defensible” assessing and scoring arrangement [17] 51.2% of the physician award-winners received their undergraduate training at one of only five UK medical schools (Table 1). These were London university medical schools, Glasgow, Edinburgh, Oxford and Cambridge. A similar pattern of concentration occurred amongst the non-physician merit award-winners; 53.0% of these were graduates of Aberdeen, Edinburgh, Glasgow, London university medical schools and Cambridge. The observation that there is a similar concentration of award-winners amongst graduates of similar medical schools, for both the physicians and non-physicians, implies that there may be common underlying non-specialty specific factors which account for the success of these doctors. The quality of undergraduate medical education may well be such a factor.

A Pearson’s Chi-Square test showed a statistically significant difference between the distributions of the medical schools of origin for physician merit award holders versus the non-physician merit award holders (*p* = 0.0002, Chi-Square 32.27). Specifically, the successful physicians were 1.6 times more likely to be graduates of London university medical schools than non-physicians. In contrast, the successful non-physicians were 3.4 times more likely to be graduates of Aberdeen university than physicians. As the top ten medical schools of origin for the physicians includes London, Oxford and Cambridge then in this instance the prestige and good quality of medical education would seem to coincide in these universities [18]. Interestingly and in contrast, the high ranking of Aberdeen medical school amongst non-physician merit award holders implies that a prestigious medical school alone is not as dominant a factor in the successful career development of non-physicians. Based on our data, a strong local or international student candidate applying to medical school who has a desire to specialize as physician might favour London, Glasgow, Edinburgh, Oxford and Cambridge medical schools, whereas a less strong student applicant who definitely did not want to specialize as physician might be wiser to prioritize Aberdeen medical school. A student who was not sure whether a physician or non-physician career pathway was preferred might consider Glasgow medical school. Thus, the rankings of medical schools that we produced in this study provide data which future prospective medical students can use to select medical schools appropriate for their ambitions. Students generally make rational decisions in the field of education [19, 20] and ranking information of this type is particularly important to an educational pathway as complex and tortuous as attempting to train to be a doctor in a particular specialty. Recent studies have demonstrated that the differences between medical schools tend to remain stable over time [21], so the guidance offered here will have valuable longevity.

Our observation of the concentration of award-winning physicians and non-physicians amongst a small number of medical schools prompted a consideration of the role of medical school size on the rankings. Specifically, after summation of the number of yearly graduates, London medical schools combine to be one of the largest medical schools in Europe. Therefore, as a proportion, London university medical schools’ graduates would probably be well represented in any essentially Eurocentric merit award schemes. To investigate this, we performed an approximate size correction to the medical school rankings by number of award-winners, as described and discussed in the methods section, using the 2019 medical school student admission numbers. Applying this to the physician award-winners rankings, London university medical schools dropped from a position of one before the approximate size correction to a position of six after size correction. A parallel effect occurred when the approximate size correction was applied to the non-physician award-winning rankings; here London university medical schools dropped in ranking from one to nine. Clearly, medical school size affects the medical school ranking. However, it is improbable that size alone can account for the concentration of clinical merit award-winners in a few medical schools; a factor related to the quality of the undergraduate medical education is also consistent with our findings.

### Physician merit awards and international medical schools

In view of the tendency of medical trainees and students to travel internationally in this era of globalization [22, 23] we also evaluated the originating medical schools of the award-winners by continent of location. Table 3 depicts the comparison of the originating medical schools for physician and non-physician merit award-winners. 91.4% of the physician award-winners originated from European medical schools whereas 91.9% of the non-physician award-winners were originally trained in European medical schools. Statistically, there was no significant difference between the continental locations of the originating medical schools for the physicians and non-physicians, in terms of their distributions, *p* > 0.05 (Chi-square test).

Our study shows a greater diversity of medical school origin amongst the lowest grade of national merit award-winners than the highest grade of national merit award-winners. Physicians with tier 1 awards came from 12 medical schools representing just one continent whereas tier 2 award-winners came from 17 medical schools representing three continents. Tier 3 award-winners originated from 61 medical schools representing six continents. These findings appear to represent a tendency to greater globalization and inclusivity effects in the lower national merit awards. The fact that the greatest concentration of IMGs, 13.3%, occurred amongst the lowest national merit awards also supports this observation. The greater number of lower grade awards and the shorter time taken to attain the lower awards than the higher awards, would naturally make such demographic trends more apparent amongst the lower merit awards. Longitudinal analyses of merit award-winners over the next decade would be valuable in accurately assessing whether this diversity trend progresses into the higher and more prestigious merit awards.

### Merit awards; undergraduate and postgraduate training of physicians and non-physicians

This research project is unique in investigating the relationship between national award-winning physicians and their originating medical schools. Specifically, little peer reviewed work has been published that investigates the effectiveness of each medical school in training their students and relates this to the future postgraduate success of each medical school’s alumni. We were only able to identify three authoritative studies [21, 24, 25]. The MedDifs study by McManus et al. [21] was the most comprehensive and included some components that were comparable to our study. The MedDifs study involved examining UK medical school performances using 50 different criteria that were either quantitative or qualitative in nature. These criteria were grouped into categories [21]:

- Selection of applicants.
- Student satisfaction.
- Curricular influences.
- Fitness to practise.
- Choice of training specialty.
- Postgraduate examination performance.
- Foundation entry scores.
- Perception of Foundation Year 1.
- Teaching/learning and assessment.
- Institutional history.

In comparing our study to the MedDifs study, we were obviously more limited in the number of factors pertinent to medical education that we considered and we followed a purely quantitative approach to the research. Unsurprisingly, McManus et al. were able to correlate their range of factors and reveal educational relationships. For example:

- Medical schools that focused on Problem Based Learning tended to produce doctors that scored lower in postgraduate exams.
- Doctors from the bigger medical schools tended to score worse in postgraduate exams.
- Medical schools that focused on self-regulated learning produced doctors that tended to perform better in postgraduate exams.

Both their study and ours shared the limitation of not being able to assess and compare medical school courses in undergraduate medical degrees. Furthermore, the MedDifs project was much more limited in its ability to identify causal relationships between its investigated educational factors.

In order to investigate the possible causalities in our presented medical school rankings for physician, non-physician and all merit award-winners (Table 1), we reviewed the histories of the UK medical schools [26–35]. We noted that all seven of the oldest medical schools in the UK, measured by establishment date, were present in our top 10 medical school rankings by award-winners. These were all established prior to 1826 and were Birmingham (1825), Manchester (1824), Aberdeen (1786), St Bartholomew’s university (1785), Glasgow (1751), St George’s London University (1733) and Edinburgh (1726) medical schools. Moreover, Oxford medical school was known to have been teaching medicine since the 12th century and Cambridge had been teaching medicine since 1524; in essence, these two medical schools had been teaching clinical disciplines before the formal establishment process had even been formed. Accordingly, it can be stated that of the top 10 medical school rankings (Table 1), *8 are the oldest medical schools in the UK.*

Furthermore, none of the more modern medical schools (established after 1999) are represented in our top 10 medical school rankings (Table 1). So, Warwick (2000), Norwich (2000), Peninsula (2000), Brighton and Sussex (2002), Hull York (2003), Keele (2003) and Swansea (2004) are not represented our top 10 (or top 20) medical school award-winner rankings. Whilst it may be understandable that the younger medical schools established within the last ten years may not yet have had time for their alumni to distinguish themselves to national merit award levels, it is less clear that this explanation accounts for the dearth of top 10 ranked medical schools established around the year 2000.

**Fig. 1:**
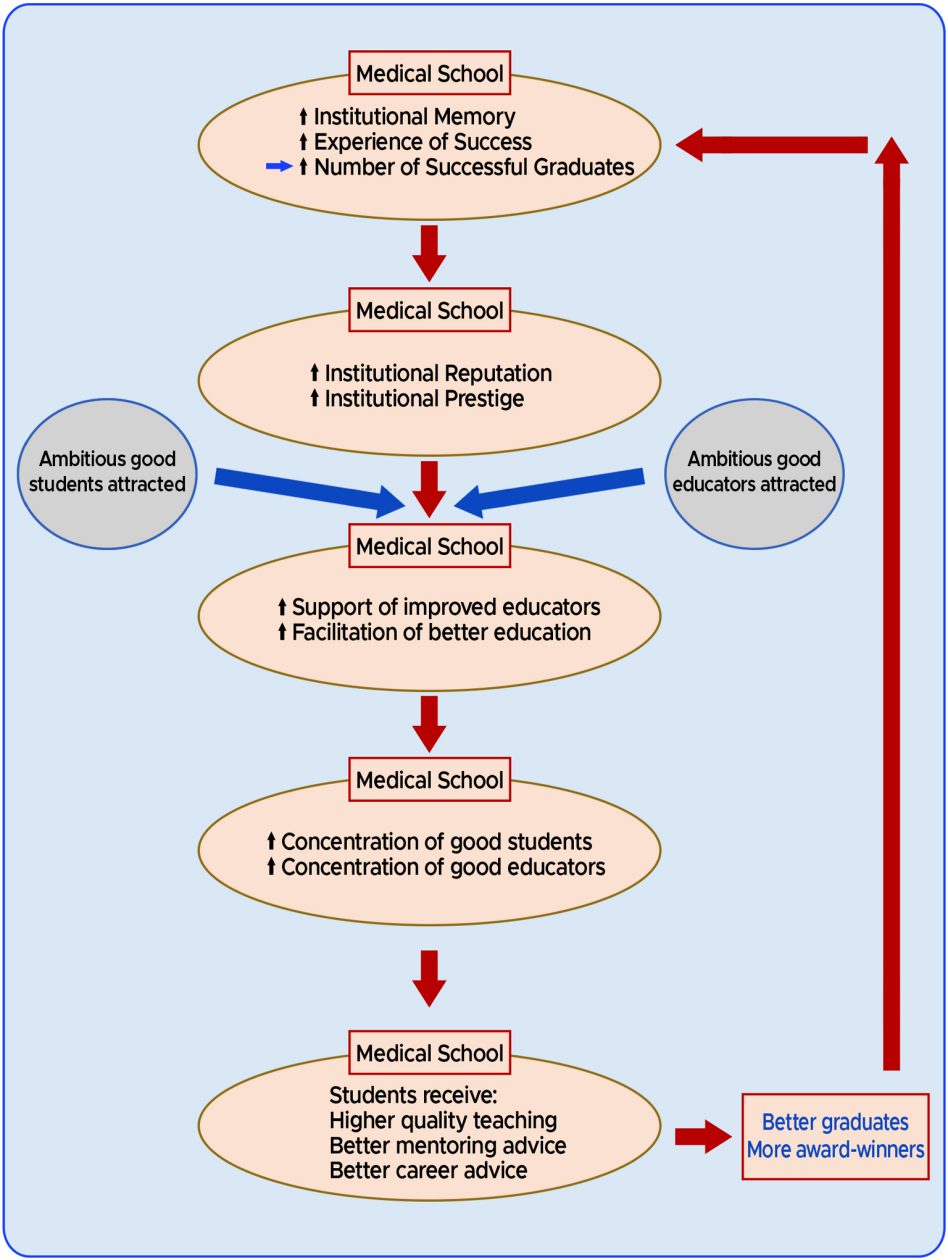
A model for the creation of award-winners. Cycles of institutional memory and experience

*In summary, our observations are consistent with at least a correlation between medical school age and the number of subsequent graduates becoming merit award-winners.* After considering the totality of the results of our research study and also accepting the previous results of the studies into UK medical school education [21, 24, 25], in Fig. 1 we reiterate a model was first described, elucidated and published earlier this year [13, 14] - a model accounting for the age-dependent differential medical school performance in creating award-winning physicians:

### Cycles of institutional memory and experience

1. As a result of their greater longevity, the **older** medical schools have **more institutional memory and experience** in education than the younger medical schools. So, the older medical schools have a greater chance of producing successful alumni before the younger schools have even been established.
2. Because of the older medical schools apparently greater number of visibly successful alumni, they may appear more prestigious and have better institutional reputations. Accordingly, **ambitious and able students** are more likely to be **attracted** to these medical schools [36].
3. These older medical schools with **greater institutional memories and experience** of producing students who achieved better postgraduate outcomes, are better placed to use this background knowledge to support and **facilitate better educators and better education**.
4. Therefore, these medical schools will accumulate a greater proportion of **more able students and more able educators**.
5. Then, the students in these university medical schools are more likely to benefit from **higher quality teaching**, better **mentoring** and better **career advice**.
6. Consequently, these medical schools are **more likely** to generate better prepared alumni who have a greater chance of becoming **merit award-winners**. *The training of these successful doctors will****add to****the institutional memory and increase the medical school’s successful experience in education and so****the cycle will repeat***.

The medical education consequences of the action of *Cycles of Institutional Memory and Experience* can be described as follows:

- An inevitable result of the operation of the adjacent cycle is that the longer established medical schools have naturally experienced more cycling during their longer existences. This causes an accumulation of an increasing number of award-winners in the medical community, from each such originating medical school.
- The differential accumulation of award-winners in the community from each medical school depends on the relative efficacy and efficiency of the cycle in each medical school. Such efficiency differences account for the ultimate medical school rankings.
- The same considerations that led to development of the Cycles of Institutional Memory and Experience can also apply to the college/departmental/faculty levels. Specifically, a department that generates merit award-winning physicians will tend to generate more such physicians in the future. In principle, this could be termed a departmental cycle of memory and experience.

Any award scheme designed and administered by human beings runs the risk of introducing biases, thus leading to overrepresentation of particular groups. Our model provides a natural explanation and mechanism for connecting excellence/success with such bias. With every cycle of our model, increasing numbers of successful graduates originating from the older universities accumulate in the UK medical community. Subsequently, such distinguished and visible alumni are more likely to be elevated to senior leadership or managerial positions. These positions would include clinical excellence/distinction award allocators. Consequently, explicit selection biases or implicit selection biases would have a tendency to favour the graduates of these same medical schools of origin - resulting in a disproportionate number of these alumni gaining awards. Ultimately, we believe our model of *Cycles of Institutional Memory and Experience*, at least in part accounts for the concurrence of appropriate success/excellence in award-winning and apparent bias in our medical school rankings. Accordingly, it seems inevitable that the effects of genuine appropriate award attainment and bias are linked and would tend to be expressed simultaneously.

In the last year there has been a reorganization of the UK national clinical excellence scheme. Specifically, in January 2022, it was announced that the latest iteration would be termed the “National Clinical Impact Awards, NCIA.” [37] The governing authority announced that the objectives of this scheme would be to:

- Widen access.
- Simplify the application process, attempting to make it more equitable and inclusive.
- Reward excellence in a wider range of activities and behaviours [38].

This new rewards scheme offers a natural test and challenge to our *Cycles of Institutional Memory and Experience* model. Our model is based on the history and epidemiology of medical education in the UK. Accordingly, an analysis of the medical schools of origin of the NCIA winners should yield rankings similar to those reported in our series of publications, assuming that there is an underlying value to the model. We look forward to testing our model in this way.

## Conclusions

Our original study uses national clinical award-winning as an outcome measure to add training and education data to the demographic description of successful doctors in Britain. Specifically, we determine and present the university medical schools which are most likely to generate award-winning physicians. We also determine and present university medical schools most likely to generate award-winning non-physicians. *This study is the first to calculate and present a ranking of university medical schools by the number of national award-winning physicians.* Accordingly, we present comparative medical school data that can be used in the rational choice of medical schools for ambitious physician inclined, non-physician inclined and undecided medical school applicants.

We demonstrate that international medical graduates are making significant contributions to good physician clinical practice in Britain, as judged by their concentration amongst the lower national merit award-winners. We provide evidence that indicates globalization and diversity of medical school origin are being reflected in the merit awards, indicating that Britain is a credible destination for ambitious medical trainees that seek national or international success.

## Data Availability

Data from this article is available upon. reasonable request to the authors. Dr Sinclair Steele is the corresponding author and will make the data available. https://www.sehd.scot.nhs.uk/publications/DC20200319SACDA.pdf. https://www.gov.uk/government/publications/accea-annual-report-2020. https://www.gmc-uk.org/registration-and-licensing/the-medical-register. https://olr.gdc-uk.org/SearchRegister.

## Abbreviations

IMG: International Medical Graduate
NCIA: National Clinical Impact Awards
CEA: Clinical Excellence Awards
DA: Distinction Awards
